# A novel peptidic inhibitor derived from *Streptococcus cristatus* ArcA attenuates virulence potential of *Porphyromonas gingivalis*

**DOI:** 10.1038/s41598-017-16522-y

**Published:** 2017-11-24

**Authors:** Meng-Hsuan Ho, Richard J. Lamont, Hua Xie

**Affiliations:** 1 Department of Oral Biology, Meharry Medical College, Nashville, TN 37208 USA; 2 Department of Oral Immunology and Infectious Diseases, University of Louisville, Louisville, KY 40202 USA

## Abstract

Periodontitis is a global health problem and the 6^th^ most common infectious disease worldwide. *Porphyromonas gingivalis* is considered a keystone pathogen in the disease and is capable of elevating the virulence potential of the periodontal microbial community. Strategies that interfere with *P. gingivalis* colonization and expression of virulence factor are therefore attractive approaches for preventing and treating periodontitis. We have previously reported that an 11-mer peptide (SAPP) derived from *Streptococcus cristatus* arginine deiminase (ArcA) was able to repress the expression and production of several well-known *P*. *gingivalis* virulence factors including fimbrial proteins and gingipains. Herein we expand and develop these studies to ascertain the impact of this peptide on phenotypic properties of *P. gingivalis* related to virulence potential. We found that growth rate was not altered by exposure of *P. gingivalis* to SAPP, while monospecies and heterotypic biofilm formation, and invasion of oral epithelial cells were inhibited. Additionally, SAPP was able to impinge the ability of *P. gingivalis* to dysregulate innate immunity by repressing gingipain-associated degradation of interleukin-8 (IL8). Hence, SAPP has characteristics that could be exploited for the manipulation of *P. gingivalis* levels in oral communities and preventing realization of virulence potential.

## Introduction

Periodontitis is one of the most common infectious and inflammatory processes of humans and is a leading cause of tooth loss^1^. Based on the National Health and Nutrition Examination Survey (NHANES), the disease affects 47% of adults aged 30 years and older in the United States with different severities^2,3^. Periodontitis is characterized by destruction of the supporting tissues of the teeth including gingiva, periodontal ligament, and alveolar bone, which is caused by uncontrolled host inflammatory responses to the pathogenic oral microbiota. Periodontal diseases and oral bacteria are also physically and epidemiologically associated with severe systemic conditions such as coronary artery disease, rheumatoid arthritis, and diabetes^4–6^. Although the current treatment for periodontitis significantly improves gingival inflammation, the relief is often temporary and recurrence of the disease is common^7,8^, due, at least in part, to incomplete elimination of the pathogens^9^, and failure to restore a health-associated microbial community^10^.

Dental plaque is a complex multispecies biofilm that is a direct precursor of periodontal diseases. Although several specific oral bacteria are associated with periodontitis, a new model of pathogenesis proposes that polymicrobial synergy among organisms in periodontal microbial communities initiates dysbiotic and destructive immune responses^11^. In this model, transition from a commensal to a pathogenic microbial community requires the colonization of keystone pathogens such as *P. gingivalis*. The colonization of oral microbial communities by *P. gingivalis* depends on its interaction and co-adhesion with antecedent colonizers of oral microbial biofilms. For example, the interaction between *P. gingivalis* and *Streptococcus gordonii*, a common inhabitant of oral biofilms, is mediated by both major and minor fimbrial subunit proteins (FimA and Mfa1)^12,13^. *P. gingivalis* interspecies interactions subsequently elevate the virulence of the entire microbial community^14–17^. This phenomenon is evident in murine models, in which low levels of *P. gingivalis* can initiate alveolar bone loss, but only in the context of a microbial community^14^. Furthermore, studies using primate models have shown that a gingipain-based vaccine reduces both the number of *P*. *gingivalis* cells and total subgingival bacterial load, as well as inhibits bone loss^18^. Several reports have also documented that the combinations of *P*. *gingivalis* and other oral bacteria such as *Treponema denticola*, *Fusobacterium nucleatum* or *S. gordonii* leads to synergistic pathogenicity in animal models^19–22^. Therefore, inhibiting the colonization and accumulation of *P. gingivalis* in a polymicrobial community is an attractive strategy for disrupting the transition of a periodontally healthy community into a destructive one.

We have previously reported that arginine deiminase (ArcA) of *S. cristatus* can repress the expression of the FimA major fimbrial subunit protein in several *P. gingivalis* strains, including strains expressing *fimA* types I, II, and III^23,24^. In an *in vitro* study, we demonstrated that *S. cristatus* ArcA significantly inhibited biofilm formation by *P*. *gingivalis* ^25^ and using a mouse model, we found that *S. cristatus* ArcA can interfere with the colonization and pathogenesis of *P*. *gingivalis* ^26^. More recently, we showed that an 11-mer peptide with the native sequence of ArcA (NIFKKNVGFKK, molecular-weight 1322.62), and theoretical pI 10.48, which we designated as SAPP (Streptococcal-derived Anti-*P. gingivalis* Peptide), represses the expression of several well-established *P. gingivalis* virulence-associated genes including *fimA*, *mfa1*, *rgpA/B*, and *kgp* ^27^.

In this study, we further tested whether SAPP interferes with virulence-associated phenotypic properties of *P. gingivalis*. A series of functional assays was performed to assess the impact of SAPP on bacterial growth, biofilm formation, invasion, and gingipain activity, as well as ability of *P. gingivalis* to manipulate host immune responses. Our data show that SAPP does not affect the growth rate of *P. gingivalis* strains, but inhibits its ability to form monotypic and heterotypic biofilms, and to invade oral epithelial cells, which are key events of establishing *P. gingivalis* infection. In addition, arginine- and lysine-specific activities of gingipain were reduced in *P. gingivalis* cells and its growth media in the presence of SAPP. Our findings demonstrate the potential of SAPP as a lead compound for the development of therapeutic agents designed to inhibit *P. gingivalis* colonization and pathogenicity.

## Results

### Effect of SAPP on biofilm formation of *P. gingivalis*

**Figure 1:**
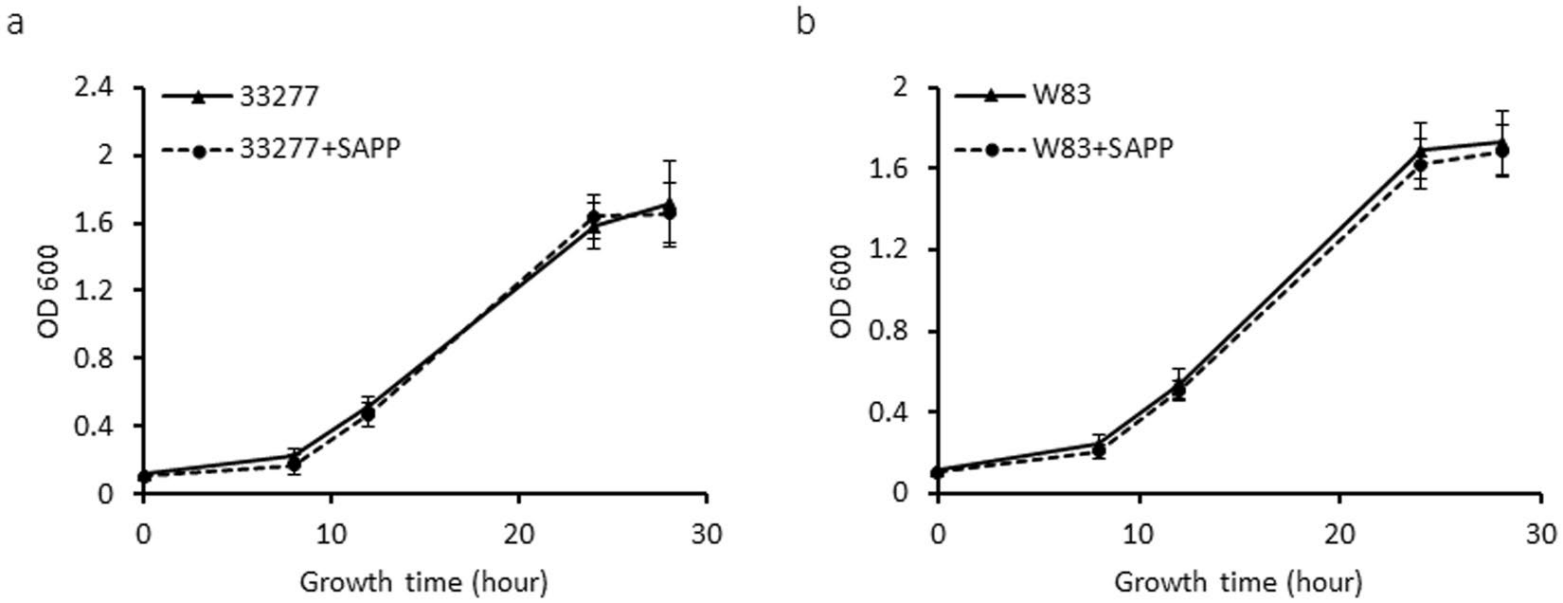
Comparison of the growth curves of *P. gingivalis* strains grown in the presence or absence of SAPP. *P. gingivalis* 33277 (**a**) and W83 (**b**) were grown in TSB media in the presence or absence of SAPP (24 μM). OD600 was measured over a period of 30 h. Curves are means of triplicate samples, with error bars representing the standard deviation.

**Figure 2:**
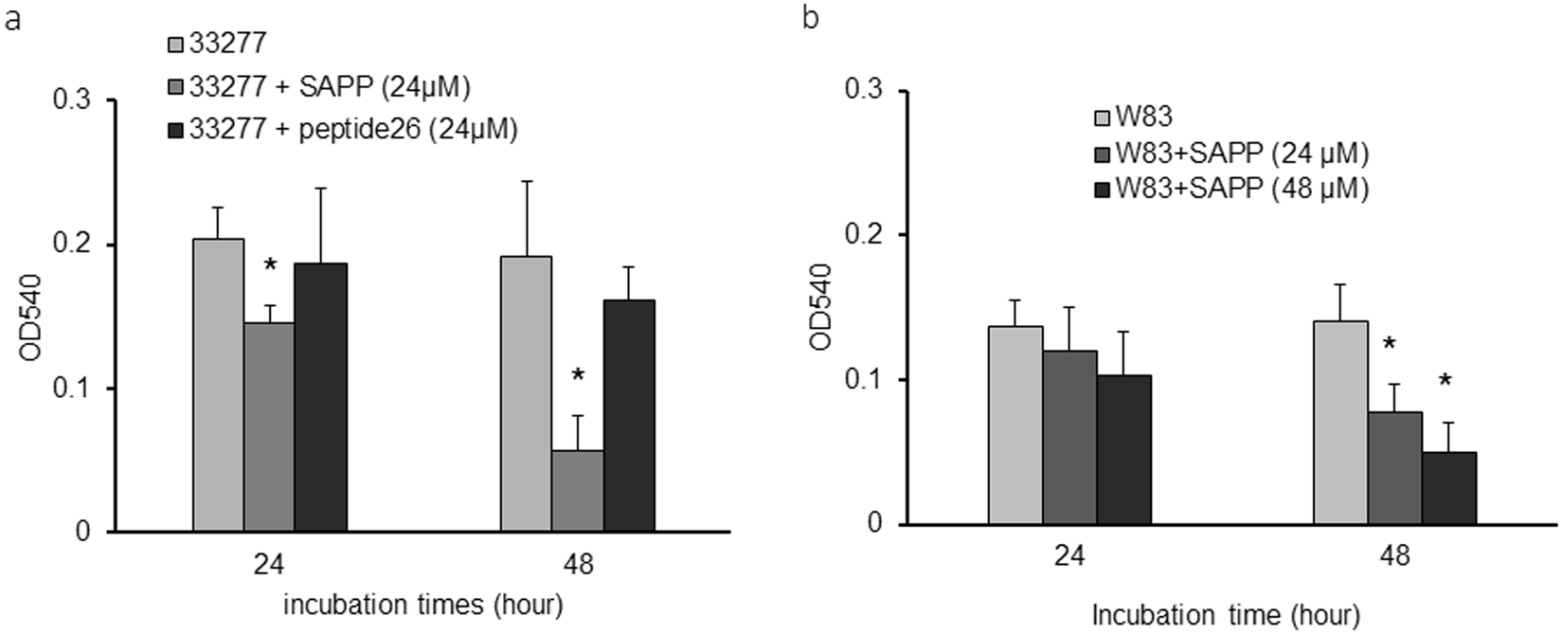
Quantitation of *P. gingivalis* attachment to a saliva-coated surface. Adherence assays were conducted in 96-well polystyrene microtiter plates. The wells were precoated with human whole saliva and inoculated with *P. gingivalis* 33277 (**a**) or W83 (**b**) grown with SAPP (24 or 48 µM) or control peptide at 37 °C anaerobically. The ability of *P. gingivalis* to attach and form microcolonies on the surface was quantified by crystal violet staining. Each bar represents the mean ± standard deviation of binding capability from three independent experiments.

*P. gingivalis* strains 33277 and W83 obtained from ATCC were selected as representatives of fimbriated, non-encapsulated and non-fimbriated, encapsulated lineages, respectively, to examine alteration of phenotypic properties of *P. gingivalis* in response to SAPP. As shown in Fig. 1a,b, growth rates of *P. gingivalis* 33277 or W83 were not significantly changed in the presence of SAPP (24 µM) compared to growth without SAPP. This observation suggests a killing-independent mechanism of SAPP action, which is in agreement with our earlier finding that SAPP represses expression and production of fimbrial proteins and gingipains in *P. gingivalis* ^28^. While the predominant niche of *P. gingivalis* is the subgingival area, the organism also colonizes supragingival plaque and oral mucosal surfaces^29^. Indeed these sites, which are exposed to the salivary fluid phase, may represent early colonization events. Hence, *P. gingivalis* strains grown with or without SAPP were then tested for their ability to attach to saliva-covered surfaces. After a 24 h incubation, an approximate 25% decrease of attachment was detected with *P. gingivalis* 33277 grown with SAPP (24 µM) compared to the control without SAPP (Fig. 2a), while the decrease in attachment reached 70% after 48 h. A control peptide (peptide26 with 11 amino acids located immediately down stream of SAPP) was also tested for its role in biofilm formation of *P. gingivalis*, and it did not significantly affect the biofilm formation. The effect of W83 on monotypic biofilm formation was also tested with the bacterial cells grown with 24 or 48 µM SAPP. The ability of W83 to form biofilms was lower than that of 33277, and an impact of SAPP on biofilm formation by W83 was not observed after 24 h. However, after 48 h a 33% and 54% reduction in biofilm formation was observed for bacteria grown with 24 or 48 µM SAPP, respectively (Fig. 2b). These results indicate that SAPP can suppress biofilm formation by both 33277 and W83, with a more efficient inhibition occurring with 33277, likely due to the involvement of fimbrial adhesins in biofilm formation by 33277.

**Figure 3:**
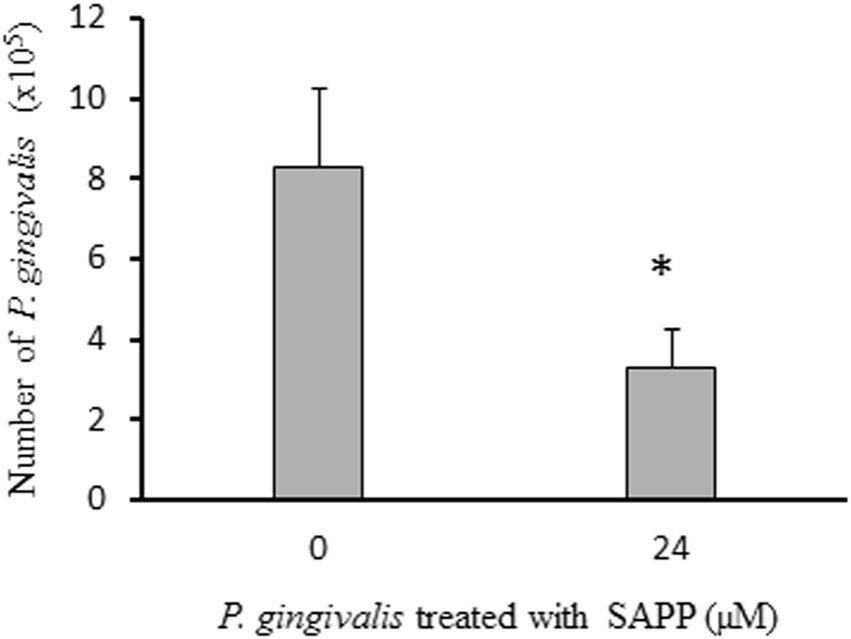
Formation of *P. gingivalis* 33277-*S. gordonii* DL1 heterotypic biofilms. *S. gordonii* DL1 biofilms were established on polystyrene surfaces coated with human whole saliva. *P. gingivalis* 33277 grown with or without SAPP (24 µM) was reacted with the DL1 biofilms for 4 h. The bound 33277 in the biofilms was quantitated using qPCR. Each bar represents the number of 33277 cells detected in the biofilms. Error bars indicate standard deviations.

*P. gingivalis* and *S. gordonii* dual-species communities are one of the best-documented examples of synergistic oral bacterial interactions. The surface molecules involved in co-adhesion are well characterized and include FimA and Mfa1 of *P. gingivalis* and streptococcal glyceraldehyde 3-phosphate dehydrogenase (GAPDH) and SspA/B^30^. We postulated that SAPP could prevent the formation of heterotypic *P. gingivalis*-*S. gordonii* biofilms by repressing the expression of *fimA* and *mfa1*. To test this, *S. gordonii* DL1 substrata were first established on saliva-coated wells and reacted with *P. gingivalis* grown with or without SAPP (24 µM). The amount of *P. gingivalis* 33277 bound to the *S. gordonii* DL1 biofilms was determined using qPCR. The number of *P. gingivalis* grown without SAPP was 2.5-fold higher than that of the bacteria grown with SAPP (Fig. 3). Not surprisingly, binding of *P. gingivalis* W83, an afrimbriated strain, to *S. gordonii* DL1 biofilms was not observed (data not shown), which further confirms the role of FimA and Mfa1 fimbriae in the interaction of *P. gingivalis* and *S. gordonii*.

**Figure 4:**
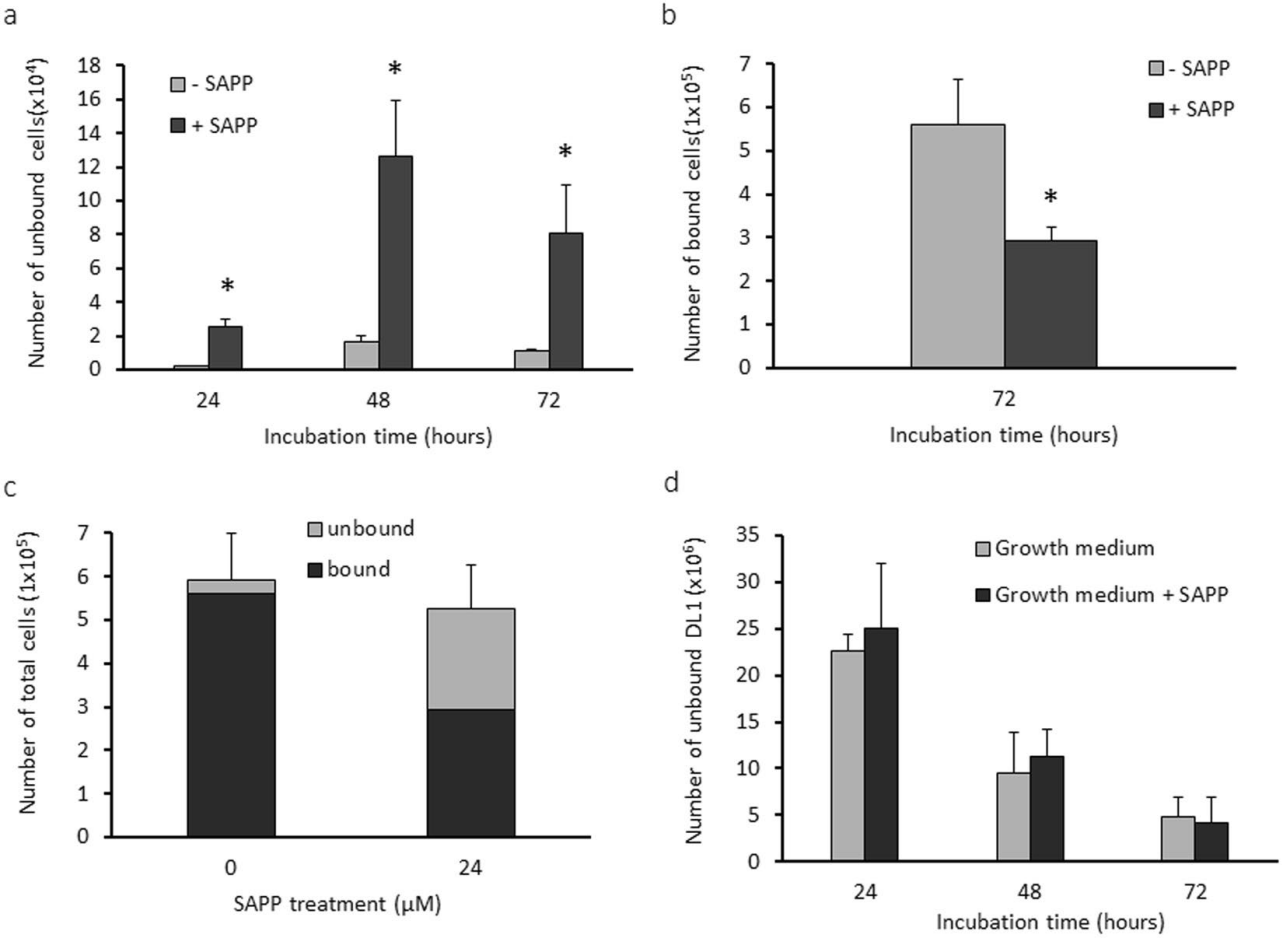
Dispersion of *P. gingivalis* from the heterotypic biofilms by SAPP. *P. gingivalis* 33277 grown without SAPP was introduced into wells of six-plates covered with *S. gordonii* DL1 biofilms to form the heterotypic biofilms. SAPP (24 µM) was then added to the wells. The numbers of *P. gingivalis* cells in the growth media (**a**), bound on *S. gordonii* biofilms (**b**), total number of 33277 in the wells (**c**), and numbers of *S. gordonii* in the growth media (**d**) were determined using qPCR. Numbers of bacterial cells in the wells with or without SAPP were compared, and an asterisk indicates a significant difference in numbers of *P. gingivalis* cells in the wells in the presence or absence of SAPP (*t* test, *p* < 0.05).

We then tested whether SAPP could reduce the numbers of *P. gingivalis* cells in established heterotypic biofilms. To do this, we first generated heterotypic biofilms of *S. gordonii*-*P. gingivalis* that were grown without SAPP. After the removal of unbound bacteria, SAPP (24 µM) was added. Planktonic *P. gingivalis* was collected three times in a 24 h interval, and the biofilm was collected after 72 h. The numbers of planktonic or sessile *P. gingivalis* were determined using qPCR. Levels of planktonic *P. gingivalis* cells were significantly increased following SAPP treatment (Fig. 4a). Consistent with this, the number of *P. gingivalis* in the heterotypic biofilms was negatively correlated with that in the planktonic phase, and introduction of SAPP to *P. gingivalis-S. gordonii* heterotypic biofilms reduced the number of *P. gingivalis* by approximately 50% after 72 h (Fig. 4b). There was no significant difference in the total *P. gingivalis* cell numbers with or without SAPP (Fig. 4c), and numbers of *S. gordonii* in the growth media did not alter in the presence or absence of SAPP (Fig. 4d). These data suggest that SAPP does not affect bacterial viability; rather, it promotes detachment of *P. gingivalis* cells in the heterotypic biofilms and inhibits their re-entry into the biofilm. Furthermore, SAPP does not impact colonization of the health-related microbiota in this model system.

### Impact of SAPP on intracellular invasion of *P. gingivalis*

**Figure 5:**
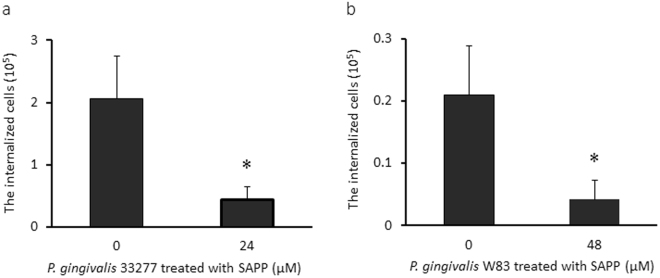
SAPP-mediated inhibition of oral keratinocyte invasion by *P. gingivalis*. Invasion of human oral keratinocytes (HOKs) by *P. gingivalis* was determined by an antibiotic protection assay. *P. gingivalis* 33277 (**a**) or W83 (**b**) were grown with 24 or 48 µM SAPP, respectively. The number of internalized *P. gingivalis* cells was represented with means ± SD of triplicates. An asterisk indicates a significant difference between invasive levels observed for *P. gingivalis* cells grown with or without SAPP (*p* < 0.05; *t* test).

The adherence of *P. gingivalis* to epithelial cells is mediated primarily by FimA and adhesive domains of gingipains^31–34^. Adherence subsequently initiates bacterial internalization of host cells^35^. Since SAPP represses FimA and gingipain production, we postulated that it may also inhibit *P. gingivalis* invasion of human oral keratinocytes (HOKs). We conducted antibiotic protection assays to compare the invasive ability of *P. gingivalis* 33277 and W83 grown with or without SAPP. Consistent with previous reports^36,37^, *P. gingivalis* W83 was much less efficient than strain 33277 in invading HOKs (Fig. 5a,b). Moreover, the invasion efficiency of 33277 grown with SAPP (24 µM) was reduced by approximate 4.8 fold compared to the control without SAPP, while a 5.2-fold difference in invasion efficiency was observed with W83 grown in the presence or absence of SAPP (48 µM). The differential potencies of SAPP on invasion inhibition of these two strains may due to different adhesins involved in their invasion process. *P. gingivalis* 33277 may mainly use its long and/or short fimbriae in the process, while W83 does not express these fimbriae and likely depends on other cell surface adhesins such as gingipains^38,39^.

### Effect of SAPP on gingipain enzymatic activity

**Figure 6:**
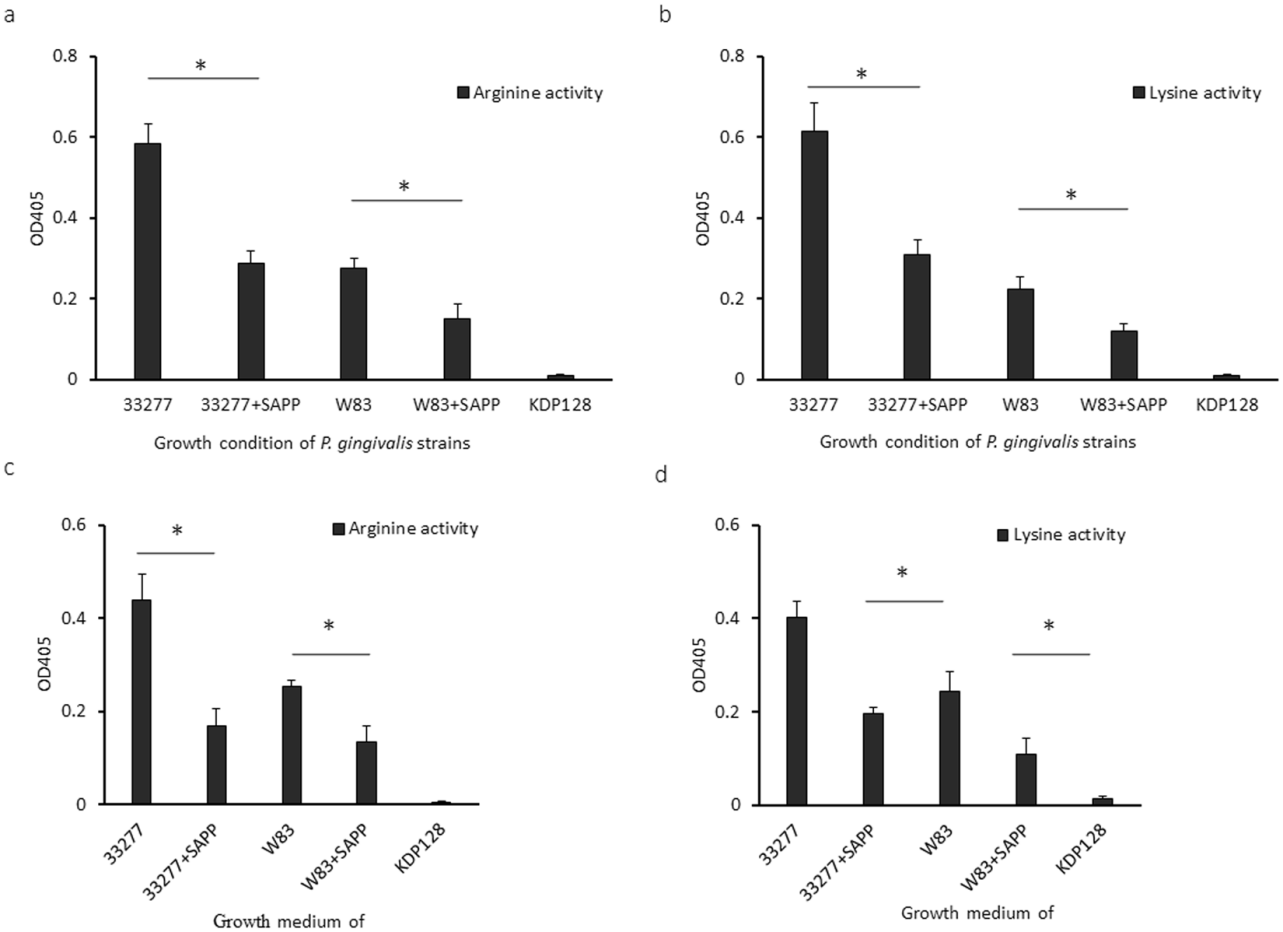
Comparison of *P. gingivalis* gingipain protease activities. Rgp or Kgp activities associated with *P. gingivalis* cells (**a,b**) or in the culture media of *P. gingivalis* (**c,d**) were tested. *P. gingivalis* 33277 or W83were grown with or without SAPP (48 µM) for 24 h, and the bacterial cells and the growth media were separated by centrifugation. Gingipain activity of KDP128 (an *rgpA* ^−^, *rgpB* ^−^, and *kgp* ^−^ mutant) was evaluated, which served as a negative control. Asterisks indicate a statistical difference of protease activity levels in *P. gingivalis* strains grown with or without SAPP (*p* < 0.05; *t* test).

*P. gingivalis* gingipains are multi-functional proteins with adhesive and catalytic domains. We have previously shown that the expression of genes encoding the arginine- and lysine-specific gingipains was repressed in *P. gingivalis* grown with SAPP^28^. Thus, we further tested and compared arginine-specific or lysine-specific activities in *P. gingivalis* strains grown with or without SAPP. An *rgpA* ^−^, *rgpB* ^−^, and *kgp* ^*−*^ triple mutant (KDP128) was used as a negative control, and as expected, no arginine- or lysine-specific enzyme activities were detected in the mutant (Fig. 6). Cell-associated arginine-specific protease activity of *P. gingivalis* 33277 grown with SAPP (48 µM) was decreased by 50%, while a 46% reduction was found in W83 under the same experimental conditions, compared to the control without SAPP (Fig. 6a). SAPP inhibited cell-associated lysine-specific protease activity of *P. gingivalis* 33277 and W83 to a similar degree (Fig. 6b). Rgp and Kgp activities in the growth media of 33277 and W83 were also monitored to determine the levels of secreted gingipains. After exposure to SAPP (48 µM), *P. gingivalis* 33277 exhibited a greater than 2.5-fold decrease in Rgp activity, and 2-fold less in Kgp activity (Fig. 6c,d). Rgp and Kgp activity in culture supernatants was also 50% lower after W83 was exposed to SAPP (48 µM). Significantly lower protease activities of Rgp and Kgp induced by SAPP may be predicted to reduce the virulence potential of *P. gingivalis*.

### Modulation of IL-8 levels by SAPP

**Figure 7:**
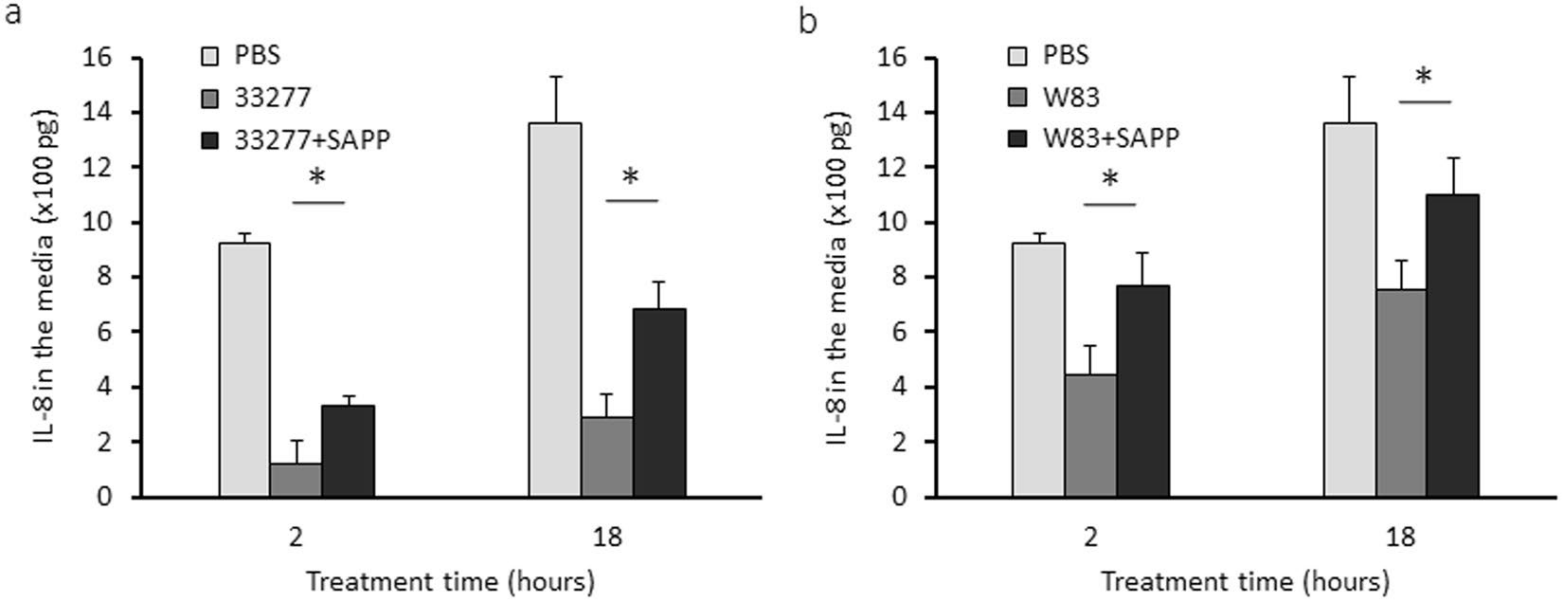
Determination of IL-8 level in the grown media of HOKs. HOKs were exposed to *P. gingivalis* 33277 or W83, at a MOI of 10 for 2 and 18 h, and the culture supernatants and HOKs were collected, respectively. IL-8 levels in the culture media (**a,b**) were measured using an ELISArray Kit. Each bar represents means of IL-8 concentration, and standard deviations were calculated from three biological replicates. Asterisks indicate statistical difference of IL-8 concentration in the culture supernatants of HOKs treated with *P. gingivalis* grown with or without SAPP (*p* < 0.05; *t*-test).

*P. gingivalis* is known to selectively impair the production of certain cytokines and chemokines, such as IL-8. Thus, we compared the accumulation of IL-8 in the growth media of HOKs exposed to *P. gingivalis* grown with or without SAPP (48 µM) using an ELISA. The results showed that the ability of *P. gingivalis* 33277 or W83 to reduce the accumulation of Il-8 was significantly diminished by SAPP (Fig. 7a,b). These data indicate that SAPP has the potential to modulate manipulation of the innate immune response by *P. gingivalis*.

## Discussion

Treatment of chronic and other forms of periodontitis usually involves physical removal of the plaque biofilm sometimes supplemented by systemic or local administration of antibiotics, especially in the cases of severe and refractory manifestations of the disease^40–42^. While the adjunctive use of antibiotics may improve the results of the mechanical therapy, meta-analyses suggest that the benefits of antibiotic treatment must be balanced against their side effects. Concerns regarding the use of antibiotics include breaking the symbiotic or mutualistic relationships between host and commensal microbiota^43^, and emerging oral bacterial resistance to antibiotics^44^. Therefore, targeting specific pathogenic bacteria, rather than non-selectively inhibiting both pathogens and commensal bacteria, has emerged as a strategic goal for maintaining a healthy oral microbiota.

Fimbrial proteins, FimA and Mfa1, and gingipains, RgpA, RgpB, and Kgp, are well-established virulence factors of *P. gingivalis*. Genes for *mfa1* and *fimA* are present in the genomes of 20 of 21*P. gingivalis* strains isolated worldwide over a 25 year time period^45^, and thus far, all tested *P. gingivalis* strains produce gingipains that are both membrane associated and secreted in soluble form^46^. The primary role of FimA and Mfa1 is to promote the bacterial attachment to various oral surfaces, which leads to biofilm formation and internalization of epithelial cells^47,48^. The gingipains include two arginine and one lysine specific cysteine proteinases (RgpA, RgpB, and Kgp)^49^. Gingipains are multi-functional proteins that have important roles in nutrient acquisition and protein processing, and can also degrade host matrix proteins and immune effector molecules^50^. Gingipains play an important role in biofilm formation, especially for the strains lacking FimA expression, through the C-terminal adhesive regions of RgpA and Kgp, or through processing profimbrillin^51,52^. Animal studies with a murine lesion model using gingipain mutants found that gingipains, Kgp in particular, are primary contributors to the formation of skin abscesses^53^. Moreover, using the same animal model, pretreatment of *P. gingivalis* with a Kgp inhibitor also decreased bacterial virulence^54^, which demonstrates an essential role of gingipains in *P. gingivalis* infection. A previous study by Wilensky *et al*. also showed that alveolar bone loss was only observed in mice orally infected with RgpA-expressing *P. gingivalis* strains^55^. Therefore, inhibition of these *P. gingivalis* virulence factors has become the focus of therapeutic strategies for prevention and treatment of periodontitis, and a long list of gingipain inhibitors has been discovered, including synthetic compounds, proteins and peptides, and extracts from plants^56^. Nonetheless, the therapeutic potential of disruption of other pathogenic processes of *P. gingivalis* is also under investigation. Several molecules derived from marine natural products were identified as inhibitors of *P. gingivalis-S. gordonii* heterotypic community formation through repression of expression of *mfa1* and *fimA* ^57^. Other promising biofilm inhibitors are small peptides representing the binding domain (BAR) of *S. gordonii* SspB, which has been shown to disrupt *P. gingivalis-S. gordonii* communities and prevent bone loss in a mouse model^21,58^. One limitation of these inhibitors is that each of them only targets a single virulence factor of *P. gingivalis*, either a fimbrial protein or gingipains. In this report, we show that SAPP derived from *S. cristatus* ArcA has a much broader spectrum and is able to impede several virulence factors simultaneously. Such multiplicity of action greatly increases the value of this peptide as a potent inhibitor capable of eliminating *P. gingivalis* strains expressing virulence factors differentially.

In this study, we have demonstrated that SAPP does not impact growth of *P. gingivalis* but significantly reduces the numbers of *P. gingivalis* cells in both monotypic biofilms and *P. gingivalis-S. gordonii* heterotypic biofilms. A reduction of *P. gingivalis* cell number may be predicted to lead to a loss of competitive edge and a disruption of polymicrobial synergy. Another significant finding is that SAPP not only inhibits *P. gingivalis* biofilm formation but also disrupts established biofilms. Although the mechanism is unclear, we speculate that SAPP disrupts attachment and inhibits re-entry of the detached bacteria to the biofilm. The dispersed *P. gingivalis* cells may be eliminated from the oral cavity due to reduced ability to attach to oral surfaces and to invade host cells. Directly targeting *P. gingivalis* attachment by SAPP should therefore stabilize a healthy microbiota and maintain homeostasis between the oral microbiota and host immune systems. Hence, it may be sufficient to prevent and treat *P. gingivalis* associated-periodontitis.

It has been reported that the expression of IL1-α, IL1-β, IL6, IL-8, IL-10, and NF-ƙB is modulated in oral epithelial cells infected with *P. gingivalis* ^59–64^. In contrast to many periodontal colonizers that induce expression of IL-8 in gingival epithelial cells, *P. gingivalis* can inhibit IL-8 production, suggesting a unique role for *P. gingivalis* in regulating epithelial cell chemokine responses^65,66^. The ability of *P. gingivalis* to modulate IL-8 levels could play a pivotal role in initiating periodontitis, as this chemokine is involved in sustaining a healthy periodontium by maintaining a gradient for neutrophil recruitment into the gingival crevice^67^. The SAPP peptide attenuate the ability of *P. gingivalis* to reduce the accumulation of IL-8 in epithelial cell culture supernatants, suggesting that SAPP is able to partially restore the impairment of host immunity by *P. gingivalis*, which may facilitate maintenance of periodontal tissue homeostasis.

In conclusion, we have assessed the inhibitory role of SAPP in biofilm formation, invasion, and gingipain activity of *P. gingivalis*. We demonstrate that a unique aspect of this peptide is that it efficiently reduced all of these virulence-associated properties of *P. gingivalis*. It should be pointed out that effective inhibitory activity of SAPP reported here is found in standard culture media and buffers, and it is not known how SAPP works *in vivo* where SAPP is dissolved in saliva and/or gingival crevicular fluid with wide range of pH^68^. In addition, more than 200 distinct bacterial species inhabit the human oral cavity of any given individual^69,70^. Future studies will be designed to determine the efficacy of SAPP under much more complex conditions. Nevertheless, the ability of SAPP to selectively disperse *P. gingivalis* from dental plaque and attenuate its virulence potential makes it an attractive agent for controlling the composition of microbial communities and maintaining a healthy microbiota.

## Methods

### Bacterial strains and growth conditions

*P*. *gingivalis* 33277 and W83 were obtained from the American Type Culture Collection (ATCC, Manassas, VA), and cultured from frozen stocks in either Trypticase soy broth (TSB) or on TSB blood agar plates supplemented with yeast extract (1 mg/ml), hemin (5 μg/ml), and menadione (1 μg/ml), and incubated at 37 °C in an anaerobic chamber (85% N_2_, 10% H_2_, and 5% CO_2_). *S. gordonii* DL1 was grown in Trypticase peptone broth (TPB) supplemented with 0.5% glucose at 37 °C under aerobic conditions.

### Peptide synthesis and activity

SAPP and a control peptide (peptide26 with 11 amino acids located immediately down stream of SAPP) were synthesized by Biomatik (Wilmington, DE) and purified with high performance liquid chromatography (HPLC) to achieve ≥ 95% purity. The purified peptide was resuspended in nuclease/proteinase-free PBS, aliquoted, and stored at −20 °C.

### Monotypic biofilm assay

Attachment of *P. gingivalis* to saliva-coated surfaces was evaluated as described previously^71^. Briefly, *P. gingivalis* strains were grown to mid-log phase (OD_600_ = 0.8) and collected by centrifugation. Bacterial cells (10^8^) were resuspended in TSB, transferred to the wells of a 96-well polystyrene plate (Corning Inc., Corning, NY) that were precoated with human whole saliva that was diluted 2 times with PBS, and incubated at 37 °C. After washing, the biofilms were stained with 1% crystal violet and destained with 95% ethanol. The absorbance of the ethanol de-staining solution at 540 nm was then determined with the Ultrospec 2100 Pro spectrophotometer (Amersham Pharmacia Biotech, Piscataway, NJ).

### Heterotypic biofilm assay

Heterotypic biofilms of *P. gingivalis* and *S. gordonii* were generated on a polystyrene six-well plate. *S. gordonii* DL1 cells (2 × 10^9^) were first incubated aerobically in saliva-coated wells at 37 °C for 3 h, and the unbound cells were removed by washing with PBS three times. *P. gingivalis* cells (2 × 10^9^) grown in TSB with or without SAPP were collected, re-suspended in ¼ TSB (1:3 TSB and PBS), added to the wells containing streptococcal biofilms, and incubated anaerobically at 37 °C for 4 h. The number of sessile *P. gingivalis* in *S. gordonii* biofilms was determined using qPCR. The bacterial cells were lysed with lysis solution (solution A; Invitrogen, Waltham, MA), and DNA was extracted using an Easy-DNA kit (Invitrogen). *P. gingivalis* cells in the biofilms were enumerated using a QuantiTect SYBR green PCR kit with *16S rRNA* gene primers of *P. gingivalis* ^25^. Standards used to determine numbers of *P. gingivalis* in the heterotypic biofilms were prepared using genomic DNA from *P. gingivalis* 33277. A fresh culture of 33277 was serially diluted in PBS and plated on TSB plates to obtain the colony forming units (CFUs) per milliliter for each dilution.

To determine whether SAPP promotes the release of *P. gingivalis* cells from the heterotypic biofilms and/or inhibits their re-entry, a modified biofilm assay was conducted. *P. gingivalis* grown without SAPP was first added to wells of six-well polystyrene plates containing *S. gordonii* DL1 and incubated for 4 h. After removing the unbound *P. gingivalis*, fresh ½ TSB (1:1 TSB and PBS) containing SAPP and gentamicin (50 μg/ml) was then added and the plates were incubated with gentle shaking under anaerobic conditions. Planktonic *P. gingivalis* were collected at three 24 h intervals, while the sessile bacterial cells were collected at the 72-h time point. Bacterial DNA was purified using an Easy-DNA kit (Invitrogen), and the *P. gingivalis* cells were quantitated using qPCR.

### Arg- and Lys-specific proteinase activities

Gingipain activities of *P. gingivalis* whole cells were measured in 96-well plates as described previously^53,72^. *P. gingivalis* 33277 or W83 were anaerobically grown in 2 ml TSB with or without SAPP to stationary phase (OD_600_ = 1.2). Bacterial cells and growth media were separated by centrifugation, and the cells were re-suspended in 2 ml ice-cold TC150 buffer (pH 8.0, 5 mM cysteine, 50 mM Tris-HCl, 150 mM NaCl, and 5 mM CaCl2). The *P. gingivalis* cell suspension (2.5 × 10^6^ cells in 2.5 µl) or 100 µl of *P. gingivalis* growth media were resuspended in TC150 buffer and mixed with 100 µl substrate solution (2 mM N-a-benzoyl-Arg-p-nitroanilide (BApNA) or N-(p-tosyl)-Gly-Pro-Lys 4-nitroanilide ace-tate salt (GPK-NA), 30% isopropanol (vol/vol), 400 mM Tris-HCl (pH 8), 100 mM NaCl, and 2 mM cysteine) (Sigma-Aldrich, St. Louis, MO). The reaction solutions were incubated at 37 °C for 4 h, and the absorbance at 405 nm was measured by a microplate reader (Bio-Rad, Hercules, CA).

### Bacterial invasion assay

The invasive ability of *P. gingivalis* was determined using an antibiotic protection assay^73^. *P. gingivalis* strains were grown in TSB for 16 h with or without SAPP to reach mid-log phase. The bacterial cells were collected by centrifugation. Human oral keratinocytes (HOKs, 5 × 10^5^) (ScienCell Research Laboratories, Carlsbad, CA) were seeded in a six-well plate. After reaction with *P. gingivalis* 33277 or W83 for 1 h, the HOKs were washed with PBS to remove unbound bacteria and then continually cultured for another 4 h in the presence of antibiotics gentamicin (300 µg/ml) and metronidazole (200 µg/ml) to eliminate extracellular bacteria. The HOKs were then washed three times with PBS and lysed with sterile distilled dH_2_O. The internalized bacteria were plated on TSB blood agar plates. The plates were incubated anaerobically at 37 °C for 7 days, and CFUs of *P. gingivalis* were enumerated.

### Enzyme-linked immunosorbent assay (ELISA)

ELISA was performed using a human IL-8 Single Analyte ELISArray Kit (Qiagen, Redwood City, CA), according to the manufacturer’s instructions. HOKs (1 × 10^5^) were exposed to *P. gingivalis* (1 × 10^6^) grown with or without SAPP for 2 and 18 h. The culture media of HOKs were collected and analyzed by ELISA. IL-8 was quantitated using a standard curve of the cytokine.

### Statistical analyses

A Student’s *t*-test was used to determine the statistical significance of differences in functions and growth rates of *P. gingivalis* strains grown in the presence or absence of SAPP. A *p* < 0.05 was considered significant. All assays were performed with three biological replicates.

## Electronic supplementary material

Supplementary Information
