# Supplementary material for: A novel peptidic inhibitor derived from Streptococcus cristatus ArcA attenuates virulence potential of Porphyromonas gingivalis
Source: Sci Rep. 2017 Nov 24;7:16217. doi: 10.1038/s41598-017-16522-y (PMC5701168; doi:10.1038/s41598-017-16522-y)
Supplement: Supplementary file 1 — Supplementary Information [file 41598_2017_16522_MOESM1_ESM.doc]

**A novel peptidic inhibitor derived from *Streptococcus cristatus* ArcA attenuates virulence potential of *Porphyromonas gingivalis***

Meng-Hsuan Ho^1^, Richard J. Lamont^2^, and Hua Xie^1^*

**Table S1. Oligonucleotide primers used in this study**

| Gene | Primer name | Primer sequences (5’-3’) |
| --- | --- | --- |
| *IL6* | IL-6 F  IL-6 R | AATCATCACTGGTCTTTTGGAG  GCATTTGTGGTTGGGTCA |
| *IL8* | IL-8 F  IL-8 R | TTCTAGGACAAGAGCCAGGAAG  GGGTGGAAAGGTTTGGAGTATG |
| *gapdh* | GAPDH-126F  GAPDH-126R | GGTGGTCTCCTCTGACTTCAACA  GTTGCTGTAGCCAAATTCGTTGT |
| *S. gordonii arcA* | SGDL1 arcA F  SGDL1 arcA R | ATGCCAGACTATCTCGAACG  CAATACCTTCATTGCGAAGC |
| *P. gingivalis*  *16s-rRNA* | PG 16s F  PG 16S R | TGTAGATGACTGATGGTGAAA  ACTGTTAGCAACTACCGATGT |
